# Posttraumatic Growth and Posttraumatic Stress Symptoms in People with HIV

**DOI:** 10.1007/s10461-022-03697-3

**Published:** 2022-06-06

**Authors:** Danni Chi, Ian de Terte, Dianne Gardner

**Affiliations:** 1 School of Psychology, Massey University, Wellington, New Zealand; 2 Clinical Psychology Centre, Ningbo Kangning Hospital, Ningbo, China; 3 School of Psychology, Massey University, Palmerston North, New Zealand

**Keywords:** Posttraumatic growth, Posttraumatic stress, HIV, Coping, Deliberate rumination

## Abstract

Receiving a diagnosis of HIV can be challenging. People with HIV (PWH) can experience high levels of distress, as well as some positive psychological changes associated with post-traumatic growth. However, the mechanisms which underlying the association of a highly stressful event (i.e., being diagnosed with HIV) and posttraumatic growth (PTG) and posttraumatic stress symptoms (PTSS) are under-explored, and this is the focus of the study. Cross-sectional survey data were provided by 77 PWH living in New Zealand. An analysis examined the roles of deliberate rumination and coping strategies as serial mediators of the associations between event centrality and PTG and PTSSs. The relationships between event centrality and PTG and PTSSs were found to be sequentially mediated by deliberate rumination and avoidance coping, but not by deliberate rumination and active coping. Further analyses explored active coping and deliberate rumination as parallel mediators, with avoidance coping as a subsequent mediator, between event centrality and PTG and PTSSs. However, these analyses were not supported. The findings indicate that the more participants appraised the HIV diagnosis as central, the greater PTG they perceived; however, the more they deliberately ruminated on it, and the more avoidance coping they adopted, the less PTG and greater PTSSs they perceived. Future studies need to explore the relationships of event centrality and coping and their associations with PTG and PTSSs.

## Introduction

Although antiretroviral therapy (ART) has prolonged life and improved the quality of life among people with HIV (PWH), receiving a diagnosis of HIV is still highly stressful. Many people have reported that receiving a diagnosis came as a shock as it came suddenly and unexpectedly [1]. Although being diagnosed with HIV may no longer be seen as a life-threatening condition, PWH face multiple stressors such as illness progression, shortened life expectancies, the side effects of medical treatment, and stigma [2]. It is not surprising that PWH have reported high levels of depression, anxiety, posttraumatic stress disorder (PTSD), and other mental illnesses in comparison to the general population [3–5]. For example, the prevalence of PTSD in PWH in the United States ranged from 10.4 to 74%, according to a paper which reviewed 33 related studies [6]. This was higher than the 3.5% incidence of PTSD in the general population of the United States [7].

In the present paper, the term ‘posttraumatic stress symptoms’ (PTSSs) is used instead of the term ‘PTSD symptoms’ in order to reduce the stigma associated with the word ‘disorder’. PTSSs include several clusters of symptoms such as intrusion, avoidance, and hyper-arousal, according to the fourth edition of the Diagnostic and Statistical Manual of Mental Disorders (DSM-IV, [8]). Another cluster of symptoms—negative cognitions and mood—was added in the DSM-5 [7]. This study followed the DSM-IV, as it is widely used in stress-related studies, which allows for comparisons with other studies.

There is growing evidence that PWH may also experience positive changes. Posttraumatic growth (PTG) refers to positive psychological changes as a result of struggling with a highly stressful event [9]. In the context of ‘posttraumatic growth’, the term ‘traumatic’ implies significant crises or highly stressful events [9]. Studies have found that between 74 and 83% of PWH reported at least one positive change [10, 11], and between 59 and 63% of PWH reported moderate or higher levels of PTG as a result of dealing with their HIV diagnosis [12–15].

PTG and PTSSs are two possible outcomes of coping with a highly stressful event. It seems logical to consider PTG and PTSSs as the opposite ends of a continuum or to expect PTG to reduce the levels of PTSSs. If so, PTG and PTSSs should be negatively associated with each other. However, studies have found positive, negative, and nonsignificant relationships between PTSSs and PTG in PWH [16–18]. One study [16] found a negative relationship between PTSSs and PTG in a sample of newly diagnosed young men with HIV in China, with an average time since diagnosis of 4.5 months. Other studies have found a positive or nonsignificant relationship between PTG and PTSSs in 114 PWH (88% of them were African American) and 110 Polish PWH, respectively, and the times since diagnosis in these two samples were 10.9 years (*SD* = 5.7) and 7.19 years (*SD* = 6.99), respectively [17, 18]. It is not clear if the inconsistencies are due to the variations in cultural background or time since diagnosis. Studies in women with breast cancer also report similar mixed findings [19–22]. Although most of the above studies are cross-sectional, and few convincing explanations for the inconsistent findings referring to the relationship between PTSSs and PTG have been put forth, the perspective that higher levels of PTG predict fewer PTSSs is not supported empirically.

PTG and PTSSs can be considered to be different outcomes of the same coping processes, and their relationship can vary under different conditions [23, 24]. Joseph and Linley [23] claim that theories of PTG should be able to explain PTSSs, but few studies have examined PTG and PTSSs together to find the shared and unique pathways associated with them in PWH.

The present study investigated PTG and PTSSs using theories developed by Tedeschi et al. [9, 25, 26] and Schaefer and Moos [24]. Although Tedeschi and Calhoun [9] provided a comprehensive description of PTG, they only focused on positive outcomes. In contrast, Schaefer and Moos [24] interpreted PTG within the stress and coping framework, which included negative as well as positive outcomes. According to these theories, a highly stressful event can trigger cognitive processing or coping strategies and lead to PTG and PTSSs [9, 24]. Several factors expected to be related to PTG and PTSSs were extracted from these two theories and related studies, including event centrality, deliberate rumination, and coping. The present study aimed to illustrate the mechanisms behind the associations of a highly stressful event (i.e., being diagnosed with HIV) and PTG and PTSSs.

Event centrality is the extent to which an event can impact on an individual’s life and identity [27]. A highly central event that changes one’s life and identity may cause psychological changes such as PTG, PTSSs, or both. Event centrality has been found to be correlated with both PTG and PTSSs [28, 29], and thus, it can be a double-edged sword [29]. However, the mechanism behind the associations between event centrality and PTG and PTSSs is not clear, which this study aimed to address.

Deliberate rumination means repetitive and intentional cognitive processing provoked by a stressful event and is different from a stable tendency to engage in habitual ruminative coping [30]. Deliberate rumination aims at understanding and problem solving and is a key contributing factor to PTG [9, 30, 31]. The positive associations between deliberate rumination and PTG have been found in people with medical conditions cross-sectionally and longitudinally [17, 20, 32]. While the rumination process aims to make sense of and derive meaning from the event, even deliberate rumination can be accompanied by distress [33, 34]. Thus, deliberate rumination is associated with both PTG and PTSSs.

Coping is defined as the constantly changing efforts to manage specific external and internal demands that are appraised as exceeding a person’s resources, and it involves both cognitive and behavioural processes [35]. A study [36] reviewed 63 studies of PWH and found that more active and less avoidance coping were associated with better physical and psychological well-being and fewer maladaptive outcomes including PTSSs. Studies in PWH have found that more use of active coping is associated with higher levels of PTG [15, 37, 38]. However, the relationship between avoidance coping and PTG is less clear. A study [38] found that active cognitive coping strategies were associated with greater PTG, whereas blaming others was negatively associated with PTG, and other forms of avoidance coping (i.e., self-blame, rumination, and catastrophising) and acceptance were not significantly correlated with PTG. Nonsignificant relationships between avoidance coping and PTG were also reported in cross-sectional studies [39, 40], as well as in a longitudinal study of women with breast cancer [41], although another study of women with breast cancer found a positive association between avoidance coping and PTG [42]. It is not clear whether the inconsistent relationships are due to the populations studied, time since diagnosis, or other factors. The present study examined the role of active and avoidance coping in the processes of PTG and PTSSs and attempted to clarify their relationships with deliberate rumination.

**Fig. 1:**
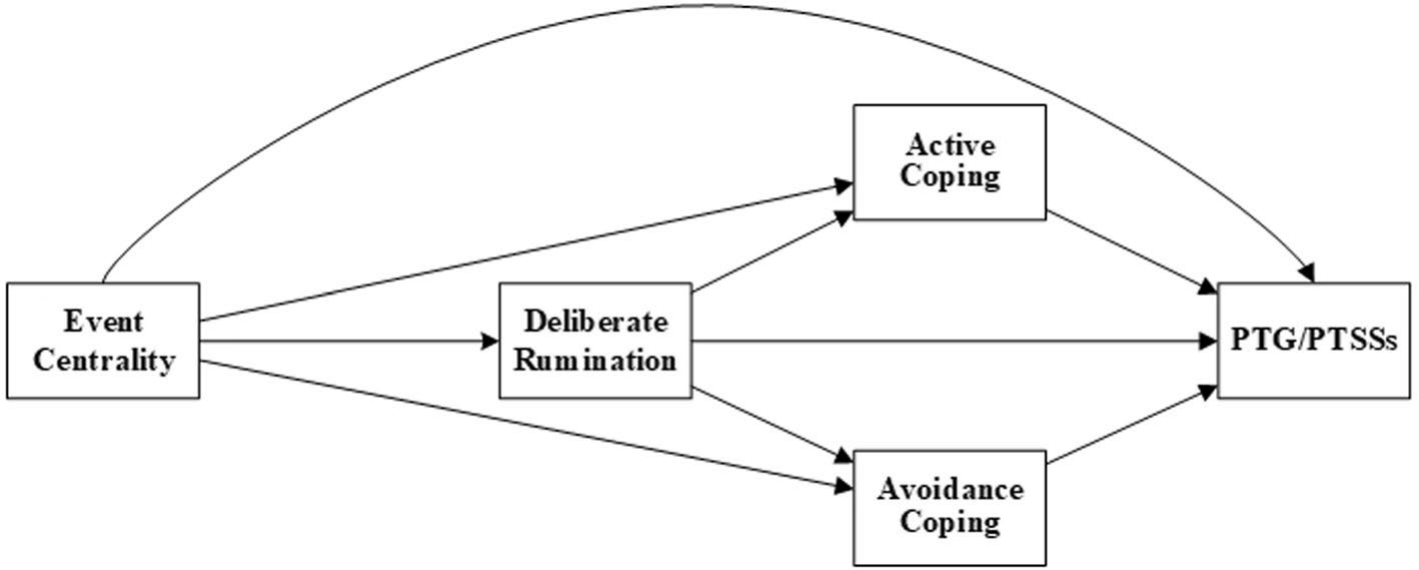
Conceptual model of deliberate rumination and active/avoidance coping as serial mediators between event centrality and posttraumatic growth (PTG)/posttraumatic stress symptoms (PTSSs)

To sum up, the associations between event centrality, deliberate rumination, active and avoidance coping and PTG and PTSSs have been reviewed. It is possible that the more central an event is, the more likely it will initiate deliberate rumination and coping processes which can affect PTG and PTSSs. Although deliberate rumination is a form of coping, it has not been as well examined as other coping strategies. It reflects attempts to process and adjust to new and disturbing information from a highly stressful experience and can help generate alternative coping strategies [43]. This study hypothesised that deliberate rumination and coping would mediate the relationships between event centrality and PTG and PTSSs (Fig. 1).

Accordingly, the hypotheses are:

- Hypothesis 1: Deliberate rumination and active coping will sequentially mediate the relationships between event centrality and PTG.
- Hypothesis 2: Deliberate rumination and avoidance coping will sequentially mediate the relationships between event centrality and PTG.
- Hypothesis 3: Deliberate rumination and active coping will sequentially mediate the relationships between event centrality and PTSSs.
- Hypothesis 4: Deliberate rumination and avoidance coping will sequentially mediate the relationships between event centrality and PTSSs.

## Methods

### Procedure

The study was approved by the Massey University Human Ethics Committee (Southern A-15/09). Information about this study including a link to the online consent form and survey was distributed on the electronic members’ boards and/or Facebook pages of the New Zealand AIDS Foundation, Body Positive, Positive Women, and the Māori, Indigenous and South Pacific HIV/AIDS Foundation, and the waiting rooms in their clinics. A paper copy of the survey was also available on request. Completion and return of the survey implied consent to participate in the study. The questionnaire took about 20 min to complete, and the recruitment period lasted for 8 months (from 10th March 2016 to 11th November 2016).

Criteria for inclusion included the following: age 18 or over, HIV positive, English fluency, and living in New Zealand. Of the 87 participants who completed the survey, three who had been diagnosed before the age of 18, and seven who had more than 40% missing data, were excluded. The final sample size was 77. All surveys were completed anonymously. At the end of the survey participants were able to choose to enter a prize draw for $100. Participants who chose to join in the draw left their contact information in a separate form which could not be connected with their survey information.

### Participants

The mean age of participants was 46.62 years (*SD* = 11.19), the mean time since diagnosis was 11.41 years (*SD* = 8.19), and the mean age of receiving the diagnosis of HIV infection was 35.21 years (*SD* = 10.01). The sample included 58 (75.3%) men, 16 (20.8%) women, and two (2.6%) transgender people. One (1.3%) participant did not provide this information.

### Measures

#### Demographics

Participants were asked to provide information on their current age (years and months), and time since diagnosis (years and months). The time-related variables also included age at diagnosis which was calculated by subtracting time since diagnosis from the current age.

#### Event Centrality

The Centrality of Event Scale 7-item version (CES-7; [27]) was used to measure the centrality of the HIV positive diagnosis during the previous 4 weeks. The CES-7 evaluates the extent to which an event impacts a person’s beliefs or becomes a central component of personal identity. Responses were on a 5-point Likert-type scale ranging from 1 (*totally disagree*) to 5 (*totally agree*). The higher the scores, the greater the event had impacted on the person’s identity. The scale score was computed as the means of items. The Cronbach’s alpha for this study was .86.

#### Deliberate Rumination

Deliberate rumination on the HIV diagnosis was measured with the Event Related Rumination Inventory (ERRI; [30]). The ERRI consists of 20 items that assess two styles of rumination: intrusive (10 items) and deliberate (10 items). In this study, only the items for deliberate rumination were assessed. Participants were asked to rate the degree to which the ruminative thoughts about their diagnosis occurred during the previous 4 weeks on a 4-point scale ranging from 0 (*not at all*) to 3 (*often*). Higher scores show more active cognitive processing. In this study, the scale score was computed as the means of items, and the Cronbach’s alpha was .86.

#### Coping Strategies

These were measured with the Brief Coping Orientations of Problems Experienced scale (Brief COPE; [44]). The Brief COPE is a 28-item self-report questionnaire modified from the original COPE [45]. Each item is rated on a 4-point Likert-type scale, ranging from 1 (*I haven’t been doing this at all*) to 4 (*I have been doing this a lot*). In this study, the Brief COPE was used to examine the coping strategies used for dealing with HIV infection in the previous 4 weeks. Principal component analysis with oblique rotation (direct oblimin) identified two factors which explained 46.23% of the total variance: active and avoidance coping. Active coping comprised 10 items, including items for active coping, planning, positive reframing, acceptance and seeking instrumental support. Avoidance coping comprised 10 items, including items for self-distraction, denial, substance use, behavioural disengagement, venting, and self-blame. The third factor only included three items and was deleted. The fourth only included two items (humour) and was also excluded. In this study, the scale scores were computed as the means of items, and the Cronbach’s alphas were .92 and .87 for active coping and avoidance coping, respectively.

#### Posttraumatic Growth

The Posttraumatic Growth Inventory-Short Form (PTGI-SF; [46]) was used to assess PTG. The PTGI-SF is a 10-item 6-point Likert-type scale, ranging from 0 (*no change*) to 5 (*very great degree of change*), selected from the original PTGI [47]. Higher scores indicate greater growth. When a single total score is desired, the PTGI-SF is a reliable substitute for the longer PTGI [46]. In this study, the scale score was computed as the mean of all items, and the Cronbach’s alpha of the present sample was .90.

#### Posttraumatic Stress Symptoms

PTSSs related to receiving the positive result of an HIV test were measured with the Impact of Event Scale-Revised (IES-R, [48]). The IES-R contains 22 items that measure subjective distress after experiencing a potentially stressful event. Respondents were asked to rate each item on a 5-point Likert-type scale ranging from 0 (*not at all*) to 4 (*extremely*) according to the frequency of symptoms they experienced [48]. The IES-R is a revised version of the original IES [49], assessing three clusters of PTSSs according to the DSM-IV [8]: intrusion, avoidance, and hyper-arousal. In this study, the event referred to receiving the diagnosis of HIV, and the IES-R was used to measure PTSSs in the 4 weeks prior to the survey. The event of receiving a positive result of an HIV test would not technically meet the criteria for a traumatic event but has been used in this way in several other studies. In this study, the total score was computed as the means of all items and the Cronbach’s alpha was .96.

### Analyses

Data analyses included descriptive statistics, bivariate analysis, hypothesis testing, and exploratory analyses. Prior to that, missing data, normality, outliers, linearity, normality, and multicollinearity were considered [50]. No significant issues were identified. Data analyses were conducted with IBM SPSS Statistics for Windows (Version 24.0). The level of significance was set to *p* < .05. The differences in levels of the continuous variables were analysed with bootstrapped independent samples *t* tests. Bivariate correlations of continuous variables were assessed using bootstrapped Pearson’s correlation. Simple mediation was examined using the SPSS plug-in, PROCESS macro Model 4; the serial mediations of the conceptual model (Fig. 1) were examined using Model 81; and the explorative serial mediations were examined using Models 80 (Version 3.4, [51]). An effect was considered significant if the confidence interval did not include zero.

## Results

### Descriptive Statistics

The levels of PTG in this study (*N* = 77, *M* = 2.69, and *SD* = 1.20) were similar to those found in the samples of PWH in the United States [52] (*N* = 112, *M* = 2.91, and *SD* = 1.36; *t* = − 1.15, *p* = .25) and the United Kingdom [53] (*N* = 38, *M* = 3.08, *SD* = 1.26; *t* = − 1.61, *p* = .11).

The levels of PTSSs in the present study (*N* = 77, *M* = 1.05, and *SD* = .88) were lower than those found in a sample of PWH in the United States [52]. It should be noted that PTSSs were measured with the IES-R in the present study but were measured with the IES in Nightingale’s study [52]. When only comparing the total score of intrusion and avoidance, the levels of PTSSs in Nightingale’s study (*N* = 112, *M* = 1.69, and *SD* = 1.21) were higher than in this study (*t* = − 4.21, *p* < .001).

In the present study, there were no significant differences between men and women in levels of PTG (*t* = − .61 and *p* = .55) and PTSSs (*t* = − 1.47 and *p* = .15), so gender was not included as a control variable in the analyses.

### Bivariate Correlations

As shown in Table 1, event centrality, deliberate rumination, active coping, and avoidance coping were positively associated with one another. Higher levels of PTG and PTSSs were correlated with greater event centrality, more deliberate rumination, and more active coping. Higher levels of PTSSs were correlated with more avoidance coping, whereas higher levels of PTG were not significantly correlated with avoidance coping.

**Table 1:** Correlation analysis of continuous variables

	1	2	3	4	5	6	7	8	9
1. Age		.45**	.69**	− .07	-.29**	− .24*	− .24*	− .19	− .12
2. Time since diagnosis			− .33**	.15	− .11	− .07	− .001	− .02	.22*
3. Age at diagnosis				− .20*	− .23*	− .19	− .25*	− .19	− .30**
4. Event centrality					.45**	.43**	.42**	.47**	.32**
5. Deliberate rumination						.76**	.40**	.58**	.41**
6. Active coping							.30**	.47**	.39**
7. Avoidance coping								.77**	− .05
8. PTSSs									.08
9. PTG									

*N* = 74–77. Results were based on 1000 bootstrap samples

*PTG* posttraumatic growth, *PTSSs* posttraumatic stress symptoms

**p < 0.01; *p < 0.05

### Hypothesis Testing

**Fig. 2:**
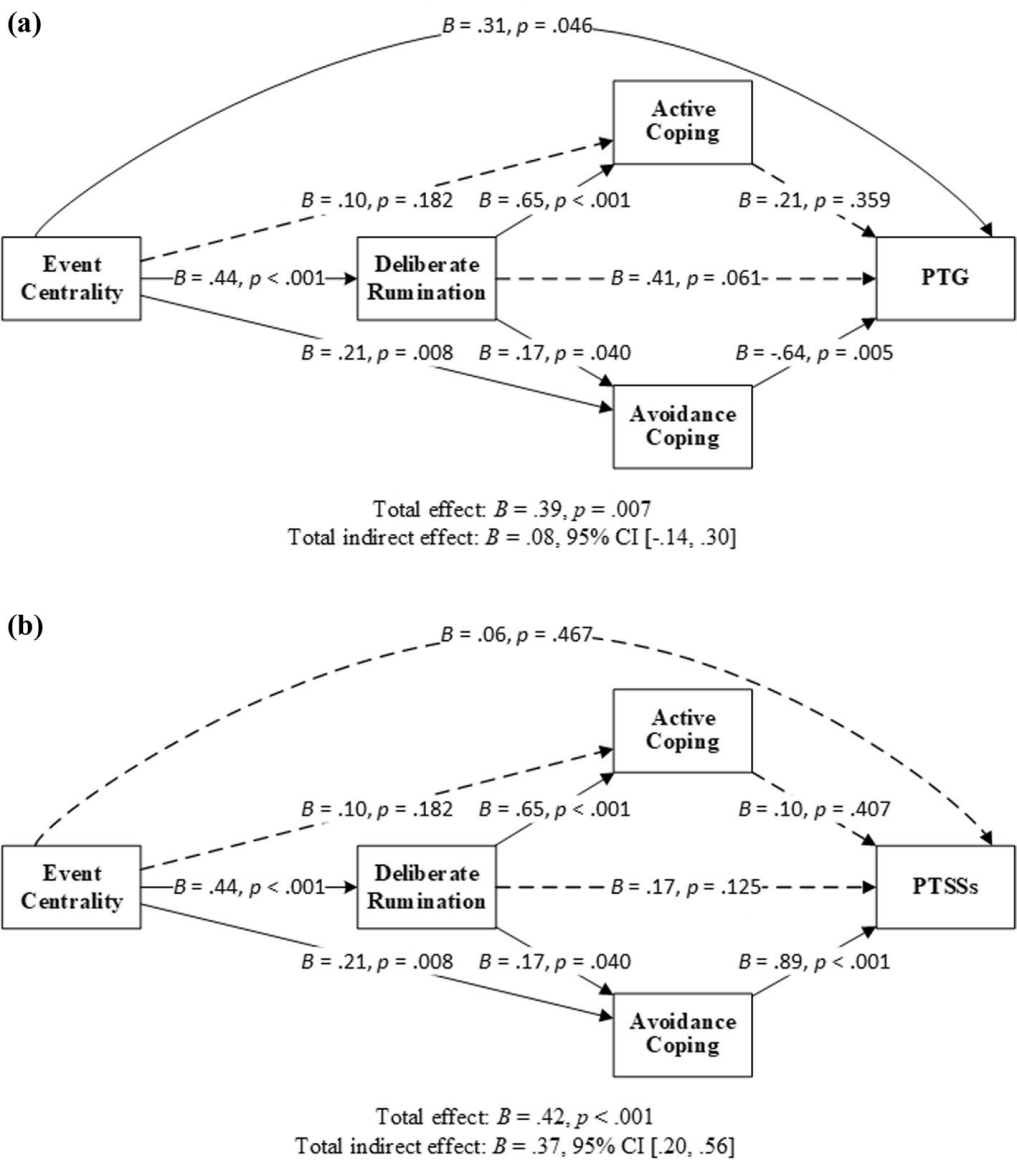
Deliberate rumination and active/avoidance coping as serial mediators between event centrality and PTG (**a**)/PTSSs (**b**). *Note N* = 77. *PTG* posttraumatic growth; *PTSSs* posttraumatic stress symptoms; *CI* confidence interval. Solid lines indicate significant paths, and dotted lines indicate insignificant paths. Results were based on 5000 bootstrap samples

Hypotheses 1 and 3 explored the roles of deliberate rumination and active coping as serial mediators between event centrality and PTG (H1) and PTSSs (H3). However, neither of these hypotheses was supported as the indirect effects of event centrality through the serial mediation of deliberate rumination and active coping on PTG and PTSSs were nonsignificant (confidence intervals included zero) (Fig. 2, Table 2). None of the specific indirect effects of event centrality through deliberate rumination or active coping on PTG or PTSSs was significant (Fig. 2, Table 2).

**Table 2:** Results of linear regression models used to examine the serial mediation of deliberate rumination and active/avoidance coping between event centrality and PTG/PTSSs

Outcome	Predictor	*B*	*SE*	*t*	95% CI	*p*
PTG	Direct					
	EC	.31	.15	2.03	[.01, .61]	.046
	Indirect					
	DR	.18	.11		[− .02, .40]	
	AcC	.02	.03		[− .03, .11]	
	AvC	− .13	.07		[− .31, − .03]	
	DR and AcC	.06	.07		[− .08, .21]	
	DR and AvC	− .05	.03		[− .10, − .001]	
	Total indirect effect	.08	.11		[− .14, .30]	
	Total effect	.39	.14	2.80	[.11, .67]	.007
	*R* = .53, *R*^2^ = .28, *F* (4, 72) = 7.03, *p* < .001					
PTSSs						
	Direct					
	EC	.06	.08	.73	[− .10, .21]	.467
	Indirect					
	DR	.08	.05		[− .01, .17]	
	AcC	.01	.02		[− .02, .04]	
	AvC	.19	.07		[.07, .34]	
	DR and AcC	.03	.03		[− .04, .10]	
	DR and AvC	.07	.04		[.002, .14]	
	Total indirect effect	.37	.09		[.20, .56]	
	Total effect	.42	.10	4.43	[.23, .61]	< .001
	*R* = .80, *R*^2^ = .65, *F* (4, 72) = 32.85, *p* < .001					

*N* = 77. Results were based on 5000 bootstrap samples

*EC* event centrality; *DR* deliberate rumination; *AcC* active coping; *AvC* avoidance coping; *PTG* posttraumatic growth; *PTSSs* posttraumatic stress symptoms; *CI* confidence interval

Hypotheses 2 and 4 explored the roles of deliberate rumination and avoidance coping as serial mediators between event centrality and PTG (H2) and PTSSs (H4). Both hypotheses were supported, as the indirect effects of event centrality through deliberate rumination and avoidance coping on PTG and PTSSs were significant (confidence intervals did not include zero; Fig. 2 and Table 2). In addition, the specific indirect effects of event centrality through avoidance coping, but not deliberate rumination, on PTG and PTSSs were significant (Fig. 2, Table 2). The positive direct effect and negative indirect effects of event centrality on PTG indicated the existence of inconsistent mediation [54].

### Explorations

**Fig. 3:**
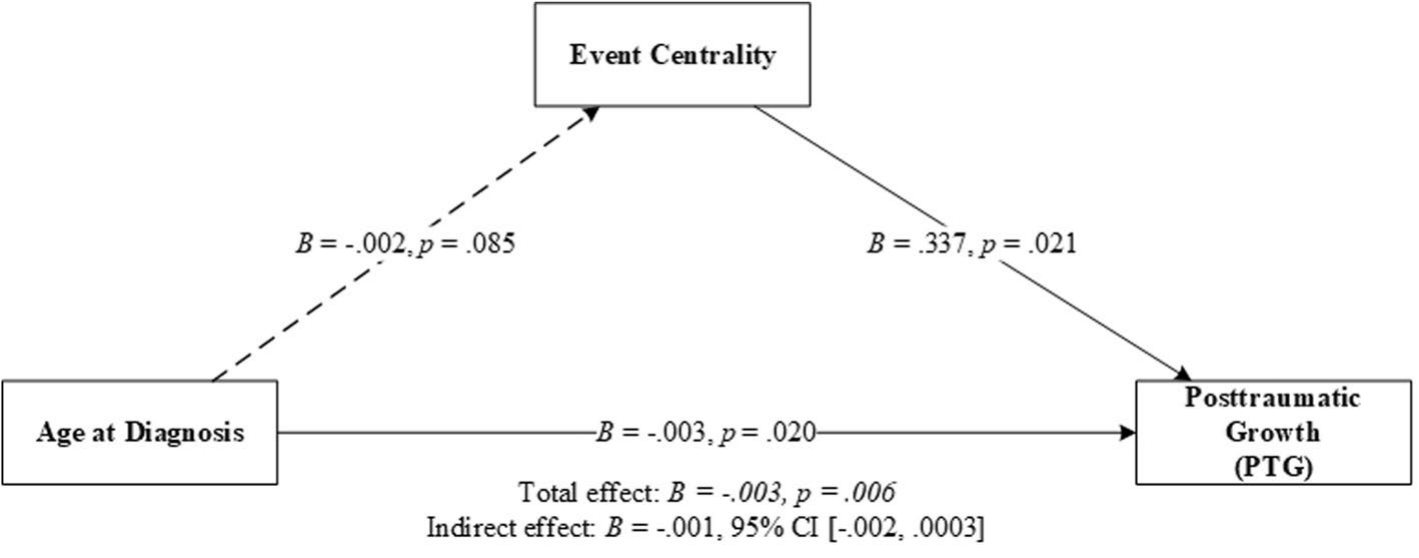
Effect of age at diagnosis on PTG as mediated by event centrality. *Note N* = 77. *CI* confidence interval. Solid lines indicate significant paths, and dotted lines indicate insignificant paths. Results were based on 5000 bootstrap samples

As shown in Table 1, age at diagnosis was negatively correlated with event centrality, deliberate rumination, and PTG. It is possible that the younger the age of diagnosis, the more central an HIV diagnosis would be, and the greater PTG would be perceived. However, this was not supported: The direct effect of age at diagnosis on PTG was significant, whereas the indirect effect of age at diagnosis through event centrality was not significant (Fig. 3, Table 3).

**Table 3:** Results of linear regression models used to examine the direct effect of age at diagnosis and mediation of event centrality on PTG

Outcome	Predictor	*B*	*SE*	*t*	95% CI	*p*
PTG						
	Direct					
	Age at diagnosis	− .003	.001	− 2.38	[− .005, − .0004]	.020
	Indirect					
	EC	− .001	.001		[− .002, .0003]	
	Total effect	− .003	.001	− 2.83	[− .006, − .001]	.006
	*R* = .32, *R*^2^ = .10, *F* (1, 72) = 8.02, *p* = .006					

*N* = 77. Results were based on 5000 bootstrap samples

*EC* event centrality; *PTG* posttraumatic growth; *CI* confidence interval

**Fig. 4:**
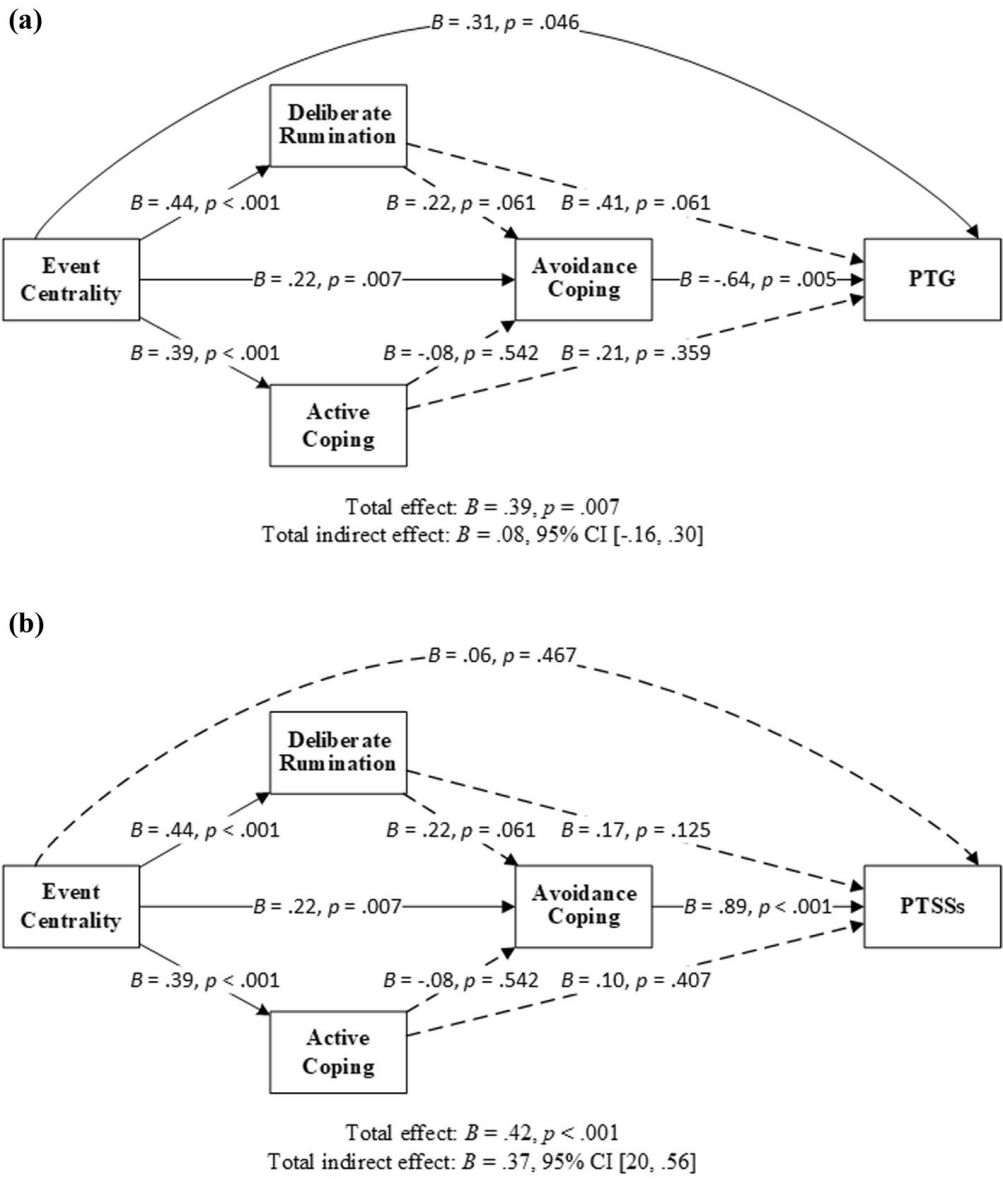
Deliberate rumination/active coping and avoidance coping as serial mediators between event centrality and PTG (**a**)/PTSSs (**b**). *Note N* = 77. *PTG* posttraumatic growth, *PTSSs* posttraumatic stress symptoms; *CI* confidence interval. Solid lines indicate significant paths, and dotted lines indicate insignificant paths. Results were based on 5000 bootstrap samples

While active coping did not act as a serial mediator with deliberate rumination in the relationships between event centrality and PTG or PTSSs, it was thought possible that active coping might act as a parallel mediator to deliberate rumination in these relationships. Further analyses explored such a possibility but were not supported, as the indirect effects of event centrality through active coping and through both active and avoidance coping on PTG and PTSSs were nonsignificant (Fig. 4, Table 4). In these explorations, only avoidance coping mediated the relationships between event centrality and PTG and PTSSs (Fig. 4, Table 4).

**Table 4:** Results of linear regression models used to examine the serial mediation of deliberate rumination/active coping and avoidance coping on PTG and PTSSs

Outcome	Predictor	*B*	*SE*	*t*	95% CI	*p*
PTG	Direct					
	EC	.31	.15	2.03	[.01, .61]	.046
	Indirect					
	DR	.18	.11		[− .03, .40]	
	AcC	.08	.10		[− .11, .29]	
	AvC	− .14	.08		[− .33, − .02]	
	DR and AvC	− .06	.04		[− .15, .0004]	
	AcC and AvC	.02	.04		[− .05, .11]	
	Total indirect effect	.08	.11		[− .16, .30]	
	Total effect	.39	.14	2.80	[.11, .67]	.007
	*R* = .53, *R*^2^ = .53, *F* (4, 72) = 7.03, *p* < .001					
PTSSs						
	Direct					
	EC	.06	.08	.73	[− .10, .21]	.467
	Indirect					
	DR	.08	.05		[− .01, .17]	
	AcC	.04	.05		[− .05, .13]	
	AvC	.19	.07		[.07, .35]	
	DR and AvC	.09	.05		[.002, .19]	
	AcC and AvC	− .03	.05		[− .12, .08]	
	Total indirect effect	.37	.09		[.20, .56]	
	Total effect	.42	.10	4.43	[.23, .61]	< .001
	*R* = .80, *R*^2^ = .65, *F* (4, 72) = 32.85, *p* < .001					

*N* = 77. Results were based on 5000 bootstrap samples

*EC* event centrality; *DR* deliberate rumination; *AcC* active coping; *AvC* avoidance coping; *PTG* posttraumatic growth; *PTSSs* posttraumatic stress symptoms; *CI* confidence interval

## Discussion

This study examined the associations between event centrality, deliberate rumination, active and avoidance coping and PTG and PTSSs in participants. The findings supported the double-edged sword role of event centrality as proposed by Boals and Schuettler [29]. Analysis identified overlapping pathways from event centrality to PTG and PTSSs: event centrality → deliberate rumination → avoidance coping → PTG or PTSSs, and event centrality → avoidance coping → PTG or PTSSs. In other words, the more participants evaluated the HIV diagnosis as central to their identity and life, the more they deliberately ruminated on it, and the more they applied avoidance coping, the less PTG and greater PTSSs they reported. Moreover, the more central the diagnosis was, and the more they used avoidance coping, the less growth and more stress symptoms they perceived. These findings suggest that the inconsistent findings for the relationship between avoidance coping and PTG reviewed in the Introduction could be caused by the presence of confounding variables such as event centrality.

In this study, active coping did not act as a sequential or parallel mediator to deliberate rumination in the relationship between event centrality and PTG or PTSSs. It appears that participants who appraised their HIV diagnosis as more central to their life, who were more likely to engage in deliberate rumination about it and who also used more active coping strategies, did not necessarily experience more PTG or fewer PTSSs.

In contrast to the positive association between active coping and mental health status reported previously [36], the present study found that more use of active coping was correlated with greater PTSSs. The relationships between coping and posttraumatic outcomes can vary according to different contexts. For example, both active and avoidance coping were less adaptive in studies where participants had a longer time since their diagnosis [36]. It is possible that the use of active coping becomes a part of life with HIV rather than a form of stress management when time since diagnosis is longer, and thus becomes less adaptive in managing PTSSs. The studies reviewed by Moskowitz et al. [36] were published between 1987 and 2005. HIV has been increasingly accepted as a chronic illness since the introduction of ART in 1996. The relationships between coping strategies and mental health outcomes have also changed. More recent studies with a range of times since diagnosis will help to clarify the relationships between coping strategies, PTSSs and PTG over time. The role of active coping strategies such as positive health behaviours as a part of PTG [10] is also worth more notice, especially in people with medical illnesses.

This study found inconsistent mediation between event centrality and PTG. On one hand, the more central the HIV diagnosis was, the more likely that participants perceived greater PTG; on the other hand, participants were also more likely to adopt avoidance coping (with or without deliberate rumination) and thus perceived less PTG. Few if any studies report the inconsistent mediation between event centrality and PTG, and replications are required.

The findings of this study suggest that deliberate rumination was also involved in the negative indirect effect of event centrality on PTG as a serial mediator prior to avoidance coping. Deliberate rumination is defined as the cognitive processing aimed at understanding highly central events and solving problems, and is different from depressive rumination [9]. The findings suggest that the effect of deliberate rumination on PTG can be more complicated than described by Tedeschi and Calhoun [9]. It is possible that deliberate rumination is a multidimensional construct. It might vary according to time since the event, valence (positive or negative), aims (searching or solving) and other factors [30, 55], and negative aspects might be associated with avoidance coping.

This study found that event centrality, deliberate rumination, and active and avoidance coping explained 28% of the variance in PTG and 65% of the variance in PTSSs. It seems that the variables extracted from Tedeschi and Calhoun [9] and Schaefer and Moos’ [24] theories were more effective in explaining the variance in PTSSs than in PTG. These findings were similar to those of two recent studies [56, 57]. One was conducted with a sample of 250 adults who experienced various adverse events in the United Kingdom and found that event centrality, intrusive and deliberate rumination, and present and future control explained 30% of the variance in PTG and 68% of the variance in PTSSs [57]. The other examined a number of variables (i.e., coping strategies, intrusive and deliberate rumination, personality traits, perceived social support, and demographics) and found that they accounted for 40% of the variance in PTG and 64% of the variance in PTSSs in a sample of 498 Turkish adults who had been exposed to stressful events. These results indicate the existence of other factors that lead to PTG and that have yet to be identified.

## Limitations

This study had some limitations. The cross-sectional design of the study meant that causality could not be determined and changes in associations between event centrality, deliberate rumination, coping, and PTG and PTSSs over time could not be examined. These relationships are likely to be reciprocal rather than unidirectional. The method of data collection limited participants to people who connected with HIV-related organisations as recruitment advertisements were distributed through these organisations. Data were also likely to be subject to self-selection bias, as individuals who were willing to participate in a study might differ from those who chose not to. In addition, the sample size was relatively small. As such, the findings might not represent the population of PWH in New Zealand. A final limitation was that some relevant information was not collected for reasons of privacy and practicality. This included education level, ethnicity, income, relationship status, and adoption of and adherence to medical and other treatments.

## Implications

This study proposed a serial mediation model to explain the associations between event centrality, deliberate rumination, coping and PTG as well as PTSSs, according to theories developed by Tedeschi and Calhoun [9] and Schaefer and Moos [24]. It identified event centrality as a double-edged sword and identified overlapping pathways between event centrality and PTG and PTSSs, through mediation by avoidance coping with or without deliberate rumination. This study revealed the inconsistent effects of event centrality on PTG: direct and positive as well as indirect and negative effects are possible. The findings suggest that the more participants appraised their HIV diagnosis as central to them, the greater PTG they perceived, but the more they deliberately ruminated on it and adopted avoidance coping, the less PTG and greater PTSSs they perceived. Replications are needed to confirm these findings.

This study also has some implications for research. Further investigation is needed into variables such as event centrality which could act as confounds in the inconsistent relationships between avoidance coping and PTG. The role of active coping, whether as a predictor, a mediator or an outcome in the relationships between event centrality, PTG and PTSSs also needs to be explored. Future studies are required to explore the multiple dimensions of deliberate rumination and their associations with PTG and PTSSs in various populations, especially among people with acute and chronic health conditions.

Clinicians need to be aware that people might experience both PTG and PTSS after their diagnosis. Clinicians may be able to support PWH to adopt less avoidance coping, in order to facilitate PTG and reduce PTSSs. While limited studies have examined interventions designed to foster PTG [58], there are indications that cognitive behavioural interventions can help decrease negative emotions and increase PTG in people with cancers [59–61]. Similarly, PWH might also benefit from cognitive behavioural interventions. Clinicians also need to note that more rumination, even deliberate rumination, can be associated with higher levels of avoidance coping and thus more PTSSs and lower PTG in PWH. It might be helpful to address deliberate rumination when it is found to be ongoing years after being diagnosed.

## Conclusions

This study examined the associations between a central event and posttraumatic outcomes, and identified the shared factors that might be associated with both PTG and PTSSs. The findings indicated that the more participants appraised an HIV diagnosis as central, the greater PTG they perceived, while the more they deliberately ruminated on it and the more avoidance coping they adopted, the less PTG and greater PTSSs they perceived. Replications, especially longitudinal ones, will help confirm these findings. Qualitative studies will help explore more dimensions of deliberate rumination and their associations with PTG and PTSSs leading to PTG.

## Data Availability

Anonymous data and related material are stored securely at Massey University under the supervision of Ian de Terte.
